# Antibacterial Photodynamic Inactivation of Fagopyrin F from Tartary Buckwheat (*Fagopyrum tataricum*) Flower against *Streptococcus mutans* and Its Biofilm

**DOI:** 10.3390/ijms22126205

**Published:** 2021-06-08

**Authors:** Jaecheol Kim, Suna Kim, Kiuk Lee, Ryun Hee Kim, Keum Taek Hwang

**Affiliations:** 1 Department of Food and Nutrition, Research Institute of Human Ecology, Seoul National University, Seoul 08826, Korea; ddeol@snu.ac.kr (J.K.); leku@snu.ac.kr (K.L.); ryunheekim@snu.ac.kr (R.H.K.); 2 BK21 FOUR Education and Research Team for Sustainable Food & Nutrition, Seoul National University, Seoul 08826, Korea; 3 Division of Human Ecology, College of Natural Science, Korea National Open University, Seoul 03078, Korea; ksuna7@mail.knou.ac.kr

**Keywords:** Tartary buckwheat, fagopyrin F, fagopyrin, photosensitizer, ROS, photodynamic therapy

## Abstract

The objective of this study was to determine reactive oxygen species (ROS) produced by fagopyrin F-rich fraction (FFF) separated from Tartary buckwheat flower extract exposed to lights and to investigate its antibacterial photodynamic inactivation (PDI) against *Streptococcus mutans* and its biofilm. ROS producing mechanisms involving FFF with light exposure were determined using a spectrophotometer and a fluorometer. *S. mutans* and its biofilm inactivation after PDI treatment of FFF using blue light (BL; 450 nm) were determined by plate count method and crystal violet assay, respectively. The biofilm destruction by ROS produced from FFF after exposure to BL was visualized using confocal laser scanning microscopy (CLSM) and field emission scanning electron microscope (FE-SEM). BL among 3 light sources produced type 1 ROS the most when applying FFF as a photosensitizer. FFF exposed to BL (5 and 10 J/cm^2^) significantly more inhibited *S. mutans* viability and biofilm formation than FFF without the light exposure (*p* < 0.05). In the PDI of FFF exposed to BL (10 J/cm^2^), an apparent destruction of *S. mutans* and its biofilm were observed by the CLSM and FE-SEM. Antibacterial PDI effect of FFF was determined for the first time in this study.

## 1. Introduction

Buckwheat plants contain protofagopyrins, which are converted to fagopyrins when the plant extract is exposed to light [1]. Both protofagopyrins and fagopyrins belong to naphthodianthrone and act as photosensitizers (PS) [1,2]. Protofagopyrins were converted to fagopyrins most rapidly when exposed to blue light (BL) and fluorescence light, and fagopyrins maintained a stable structure even when exposed to various light sources for 8 h [3]. Hypericin, one of naphthodianthrones structurally similar to fagopyrins and protofagopyrins, has been studied as a PS for various photodynamic effects including antibacterial effects [4,5,6]. Benković et al. reported the structural characteristics of fagopyrins, which are similar to hypericin [1]. Fagopyrins and hypericin have the same absorbance spectra (maximum absorbance: 590 nm) [1,3]. Also, fluorescence of fagopyrins and hypericin was detected at an excitation wavelength of 330 nm and emission wavelength of 590 nm [1,7,8,9]. However, to the best of our knowledge, photodynamic effects of fagopyrins and protofagopyrins have not yet been reported. Tartary buckwheat flowers (TBF) are richer in fagopyrins (protofagopyrins) than the other parts of the buckwheat plant (e.g., stems, leaves, flowers, groats, and hulls), regardless of the cultivar type [1,3,7,9]. Six fagopyrins (A–F) were reported to exist in buckwheat plants with fagopyrins A, E, and F identified [1,8]. In addition, Kim and Hwang reported that fagopyrin F (FF) accounted for more than 93% of total fagopyrins in TBF extracts exposed to light [9]. Given these results, in this study, we prepared FF-rich fraction (FFF) separated from the TBF extract exposed to lights and further determined photodynamic effect of the FFF.

**Figure 1:**
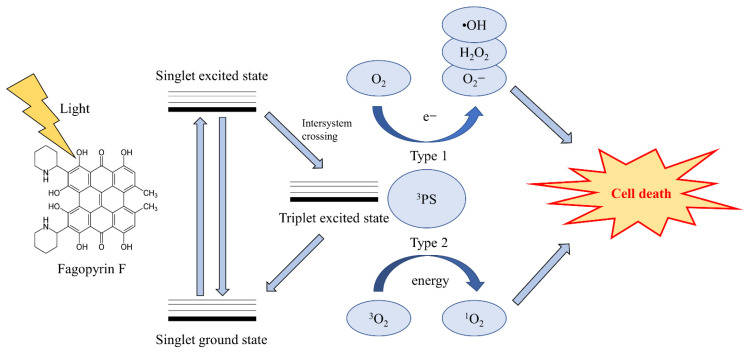
Structure of fagopyrin F and schematic illustration of photodynamic therapy.

Photodynamic therapy (PDT) is a clinical treatment against harmful microorganisms, tumors, viruses, and parasites and is based on the photochemical reaction of PS [10,11]. PS molecules absorb light of a specific wavelength initiating reactive oxygen species (ROS) production, which leads to selective cellular or tissue destruction (Figure 1) [10,11,12]. After exposure to light, energy from the triplet excited state of the PS is transferred to two ROS production mechanisms [10,11,12,13]. In the type 1 ROS photodynamic mechanism, the PS transfers hydrogen or electrons to biomolecules from its surroundings. This process initially produces ROS in the form of superoxide anion radicals (O_2_^•−^), which further generates other ROS molecules, such as hydrogen peroxide and hydroxyl radicals, inside the cell [10,11,12,13]. In the type 2 ROS photodynamic mechanism, energy from the triplet excited state of the PS is directly transferred to oxygen molecules in the ground energy state (^3^O_2_). This process produces singlet oxygen (^1^O_2_), which exhibits strong oxidative properties [10,11,12,13].

One of the most widely studied PDT applications is antibacterial photodynamic inactivation (PDI), which can be applied to several bacterial species. Among possible treatment sites for PDI, oral cavity is exposed outside, not inside the human body; thus, PDI can be directly applied to the oral cavity to inactivate various harmful bacteria. Since the PDI method for the oral cavity is simple, and PDI does not cause antibiotic resistance to harmful bacteria in the oral cavity, PDI has been studied fairly extensively for eliminating harmful bacteria in the oral cavity [14,15,16]. *Streptococcus mutans* is a gram-positive bacterium that causes dental erosion by forming biofilm [16]. Biofilm, a complex of bacteria and their secondary metabolites (e.g., sugars, acids, and glucans), forms a layer on the surface of the teeth [16]. Various PS have been used to inactivate harmful oral bacteria. Among them, curcumin and hypericin originated from food sources, are typical PS against harmful oral bacteria [4,17,18,19]. PDI of curcumin or hypericin as a PS destroyed most *S. mutans* (over 99%) and inhibited their biofilm formation [4,17,18]. Application of PDI to *S. mutans* has been studied in terms of biofilm inactivation and destruction, and the treatment effect of PDI has been mainly confirmed using spectrometer and microscope techniques such as confocal laser scanning microscopy (CLSM) and field emission scanning electron microscope (FE-SEM) [14,16,20,21,22,23,24,25,26].

The purpose of this study was to evaluate photosensitizing ability of FFF separated from TBF extract by measuring the amount of ROS produced after exposure to lights and to investigate the PDI effect of FFF against *S. mutans* and its biofilm.

## 2. Results and Discussion

### 2.1. ROS Production by FFF

**Figure 2:**
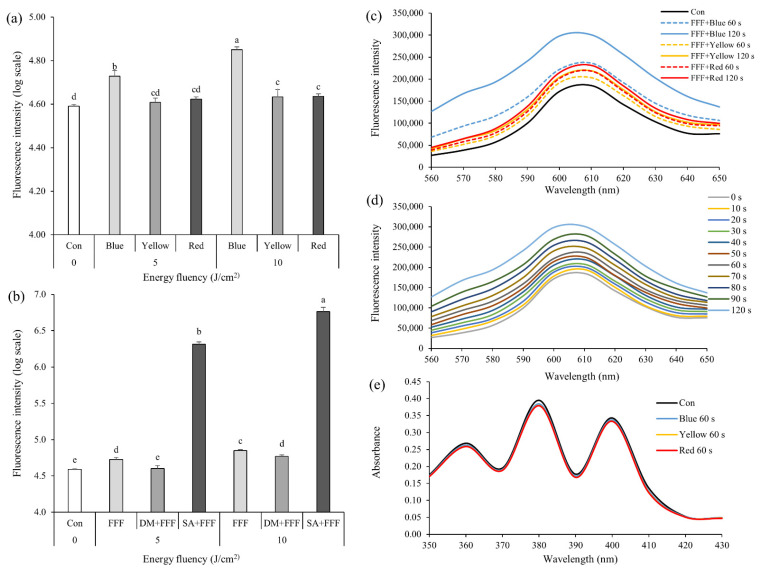
Production of reactive oxygen species (ROS) by fagopyrin F-rich fraction (FFF; final concentration of 5 μg/mL) from Tartary buckwheat flower extract after exposure to lights. (**a**) Intracellular ROS production in *Streptococcus mutans* treated by FFF with different lights. (**b**) Intracellular ROS production in *S. mutans* treated by FFF with blue light (5 or 10 J/cm^2^) with different radical scavengers. DM: D-mannitol (final concentration of 100 mM). SA: sodium azide (final concentration of 100 mM). (**c**) Superoxide production in *S. mutans* treated by FFF with different lights. (**d**) Superoxide production in *S. mutans* treated by FFF with blue light. (**e**) Singlet oxygen production treated by FFF with different lights. (**a**–**e**) Con: control with FFF without irradiation. (**a**,**b**) Different small letters indicate significant differences (*p* < 0.05; one-way ANOVA and Duncan’s multiple range test). (**a**,**b**) Bars are means ± standard deviations (*n* = 3). (**c**–**e**) Data lines are means (*n* = 3).

Intracellular ROS productions in the *S. mutans* suspensions added with FFF when exposed to BL (450 nm), yellow light (YL; 590 nm), and red light (RL; 660 nm) were shown in Figure 2a. The fluorescence intensity of the *S. mutans* suspension with FFF exposed to BL (5 or 10 J/cm^2^) was significantly higher than that of YL or RL (Figure 2a), indicating that BL was an appropriate light source to produce ROS by FFF. The fluorescence intensity of the *S. mutans* suspension added with FFF along with D-mannitol when exposed to BL (5 or 10 J/cm^2^) was significantly lower than that with FFF only (Figure 2b), suggesting that FFF exposed to BL might produce hydroxyl radicals, which might be then scavenged by D-mannitol.

On the other hand, when the *S. mutans* suspension with FFF along with sodium azide (NaN_3_) was exposed to BL (5 or 10 J/cm^2^), the fluorescence intensity did not decrease as the singlet oxygen might be scavenged by sodium azide, and the fluorescence intensity rapidly increased during exposure to BL (Figure 2b). This result implies that the fluorescence intensity increases by binding 2′,7’-dichlorofluorescin diacetate (DCFH-DA) to azide radical (N_3_^•^) derived from NaN_3_ rather than singlet oxygen produced by FFF [20,27]. That is, when FFF was exposed to BL, singlet oxygen might not be generated or the amount of singlet oxygen might not be enough to be combined with DCFH-DA.

Intracellular superoxide productions in the *S. mutans* suspensions added with FFF when exposed to BL, YL, and RL were shown in Figure 2c. The fluorescence intensity of the *S. mutans* suspension with FFF when exposed to YL or RL for 120 s was slightly higher than the control (Figure 2c), whereas the intensity of the FFF suspension exposed to BL for 120 s increased in a time-dependent manner (Figure 2d). Superoxide production by FFF exposed to BL was higher than that exposed to YL or RL. These results were consistent with those measured by DCFH-DA.

When 9,10-anthracenediyl-bis(methylene)dimalonic acid (ABDA) added with FFF was exposed to BL, YL, or RL, its absorbance, whose reduction indicates more singlet oxygen production, differed little from that of the control, regardless of the wavelength (Figure 2e). However, the previous study observed a rapid decrease in the absorbance when ABDA added with PS such as methylene blue, toluidine blue, and rose bengal was exposed to light [21,28], suggesting these PS may very effectively produce singlet oxygen following the type 2 ROS production mechanism. Thus, our result suggests that FFF exposed to light little produces singlet oxygen.

In summary, ROS production mechanism of FFF exposed to lights might be type 1. Also, type 1 ROS were more produced when FFF was exposed to BL than YL and RL. Therefore, subsequent PDI experiments were conducted using BL.

### 2.2. Photodynamic Inactivation of Planktonic S. mutans

**Figure 3:**
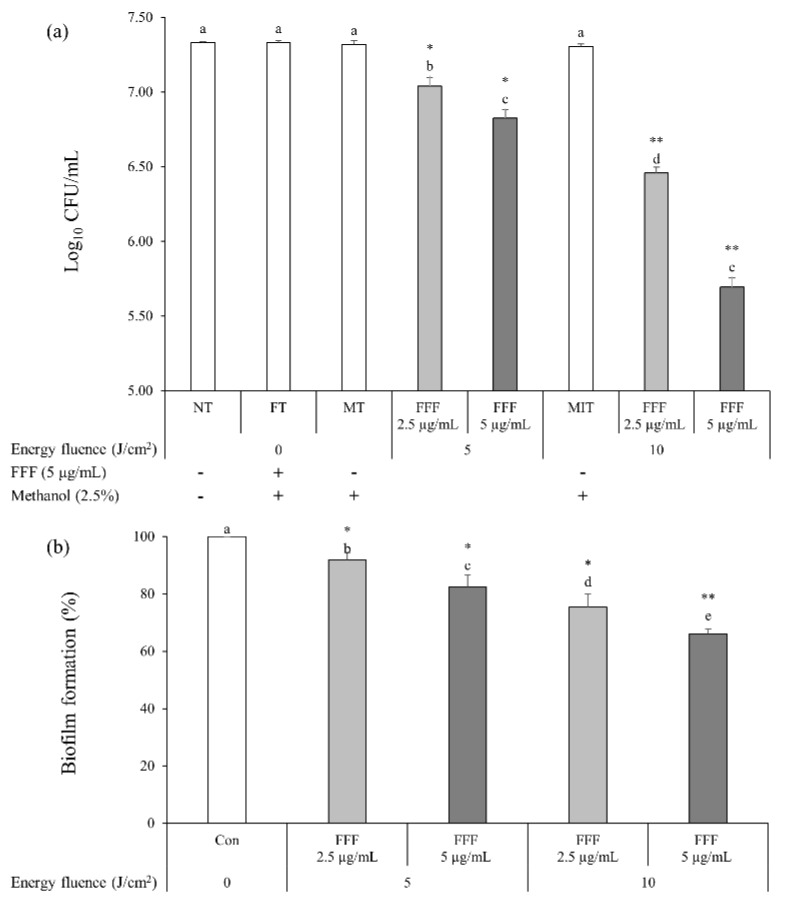
Photodynamic effect of fagopyrin F-rich fraction (FFF) from Tartary buckwheat flower extract against *Streptococcus mutans* and its biofilm. (**a**) Inactivation of *S.mutans* treated with FFF exposed to blue light (BL). NT: *S. mutans* suspension with no FFF treatment nor irradiation. FT: *S. mutans* suspension treated with FFF (final concentration of 5 µg/mL) without irradiation. MT: *S. mutans* suspension in 2.5% methanol (the same concentration of methanol in the suspension treated with FFF at the final concentration of 5 µg/mL) with no FFF treatment nor irradiation. MIT: *S. mutans* suspension in 2.5% methanol without FFF, which was exposed to BL (10 J/cm^2^). (**b**) Biofilm formation of *S. mutans* treated with FFF exposed to BL. Con: control with FFF (final concentration of 5 μg/mL) without irradiation. (**a**,**b**) Different small letters indicate significant differences (*p* < 0.05; one-way ANOVA and Duncan’s multiple range test). *, ** Significant difference compared to FT (**a**) or Con (**b**) (*p* < 0.05, *p* < 0.01; independent *t*-test). Bars are means ± standard deviations (*n* = 3).

The colony forming unit (CFU) of *S. mutans* did not significantly decrease after FT (*S. mutans* suspension treated with FFF (final concentration of 5 µg/mL) without irradiation), MT (*S. mutans* suspension in 2.5% methanol (the same concentration of methanol in the suspension treated with FFF at the final concentration of 5 µg/mL) with no FFF treatment nor irradiation), or MIT (*S. mutans* suspension in 2.5% methanol without FFF, which was exposed to BL of 10 J/cm^2^) treatment compared to NT (*S. mutans* suspension with no FFF treatment nor irradiation) (Figure 3a), suggesting 5 µg/mL FFF or 2.5% methanol (final concentration) in the suspension without BL exposure might not kill *S. mutans*. The BL exposure (10 J/cm^2^) of the suspension without FFF (MIT) did not affect *S. mutans* viability in this study, although BL (400–450 nm) is known to have antibacterial effects [20]. Paschoal et al. reported that *S. mutans* was not killed when exposed to BL (450 ± 30 nm; 24, 48, or 72 J/cm^2^) without PS treatment [17]. Moreover, *S. mutans* viability did not decrease when exposed to BL (405 nm; 25.4 J/cm^2^) without a PS [18]. In this study, *S. mutans* was exposed to BL (5 or 10 J/cm^2^), in which energy fluences were lower than in the previous studies [17,18]. Therefore, it is certain that BL (5 and 10 J/cm^2^) tested in this study does not affect *S. mutans* viability.

When the *S. mutans* suspension added with FFF was exposed to BL (5 and 10 J/cm^2^), CFU in the PDI treatment groups were significantly lower than the NT and FT (Figure 3a). CFU in the *S. mutans* suspension added with FFF when exposed to BL decreased in a dose-dependent and energy fluence-dependent manners. In this study, when *S. mutans* suspension added with FFF (5 μg/mL) was incubated for 10 min and then exposed to BL (450 nm; 10 J/cm^2^), *S. mutans* viability decreased by 97.6% compared to the FT. Lüthi et al. reported that when *S. mutans* was incubated with hypericin (10 μg/mL) for 30 min and then exposed to BL (400–505 nm) with energy fluence of 128.4 and 256.8 J/cm^2^, *S. mutans* viability decreased by 99.2 and 99.998%, respectively, compared to the control [4]. Paschoal et al. also reported that when *S. mutans* was incubated with curcumin (2 mM; 736.8 μg/mL) for 1 min and then exposed to BL (450 ± 30 nm; 24 J/cm^2^), *S. mutans* viability decreased by 99.13% compared to the control [17]. However, considering that PS concentrations and energy fluences in the previous studies were higher than those in this study, FFF may have comparable potency to kill *S. mutans* to other PS. On the other hand, Ribeiro et al. reported that when *S. mutans* was incubated with riboflavin (40 μg/mL) for 10 min and exposed to BL (455 ± 20 nm; 32.4 J/cm^2^), *S. mutans* viability decreased by 77.5% compared to the control [19], in which a smaller decrease in viability of *S. mutans* was observed although a higher PS concentration and a higher energy fluence were applied than in this study.

### 2.3. Effects of PDI Treatment of FFF on S. mutans Biofilm Formation

*S. mutans* biofilm formation in the PDI treatment groups was significantly lower than that in the control (Figure 3b). The *S. mutans* suspension added with FFF (5 µg/mL) exposed to BL (10 J/cm^2^) had the highest inhibition in the biofilm formation among the PDI treatment groups. Inhibition rates of biofilm formation with the PDI treatments of FFF at 2.5 µg/mL exposed to BL of 5 J/cm^2^ and FFF at 5 µg/mL exposed to BL of 10 J/cm^2^ were the lowest (11.6%) and the highest (34.0%), respectively, compared to the control (Figure 3b). In this study, the PDI treatment with a higher concentration of FFF formed less biofilm at the same energy fluence (Figure 3b). However, the PDI treatment with FFF at 2.5 µg/mL exposed to BL of 10 J/cm^2^ formed a significantly less biofilm than the PDI treatment with FFF at 5 µg/mL exposed to BL of 5 J/cm^2^ (*p* < 0.05; Figure 3b). These results suggest that increased energy fluence might be more efficient than increased FFF concentration in inhibiting *S. mutans* biofilm formation.

In this study, we confirmed for the first time that FF in the FFF, when exposed to BL, exerts antibacterial and antibiofilm effects in *S. mutans*. As previously stated, inactivation of *S. mutans* and biofilm formation by PDI using hypericin was reported [15]. It has been also reported that hypericin has a PDI effect on several gram-positive bacteria [4,15]. Thus, further studies are warranted to confirm the PDI effects of FFF on other harmful gram-positive bacteria.

### 2.4. Visualization of Antibiofilm Effects of FFF by CLSM

**Figure 4:**
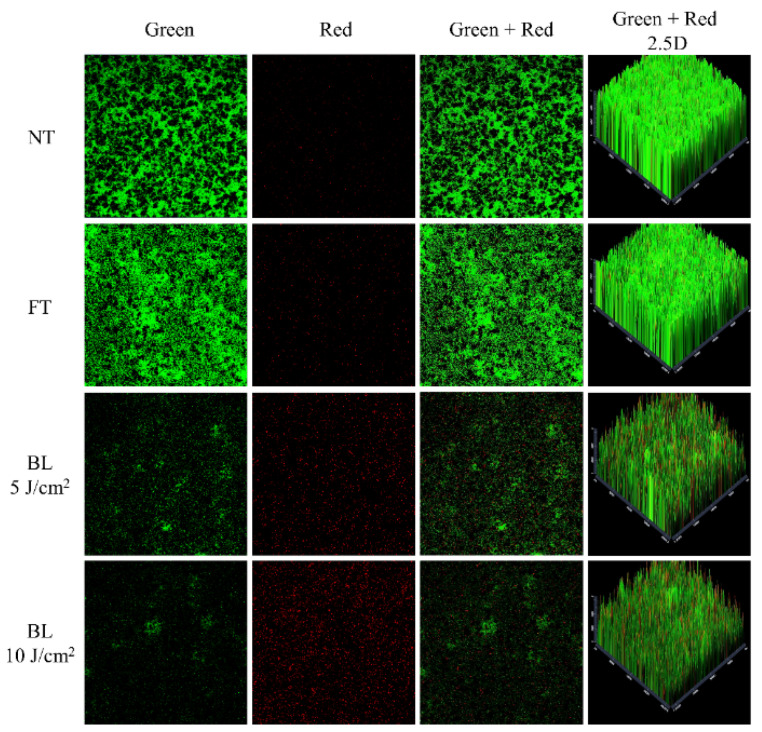
Confocal laser scanning microscopy images of *Streptococcus mutans* biofilm treated with photodynamic therapy of fagopyrin F-rich fraction (FFF; final concentration of 5 μg/mL) exposed to blue light (BL). NT: *S. mutans* biofilm with no FFF treatment nor irradiation. FT: *S. mutans* biofilm treated with FFF without irradiation.

CLSM was performed to visualize the PDI effects of FFF against *S. mutans* biofilms (Figure 4). SYTO 9 and propidium iodide dyes were used for staining living cells green and dead cells red, respectively, and confirmed whether bacterial death occurred after the PDI treatment of FFF [29]. The fluorescence images of the NT and FT showed dense green staining indicating live bacteria (Figure 4). However, when the energy fluence increased, this green staining gradually dimmed and red staining gradually increased, indicating bacterial death. These results suggest that the PDI treatment of FFF may be effective in destructuring *S. mutans* biofilms. These observations were similar to CLSM image color changes from previous PDI studies using other PS [21,30].

### 2.5. Visualization of Antibiofilm Effect of FFF by FE-SEM

**Figure 5:**
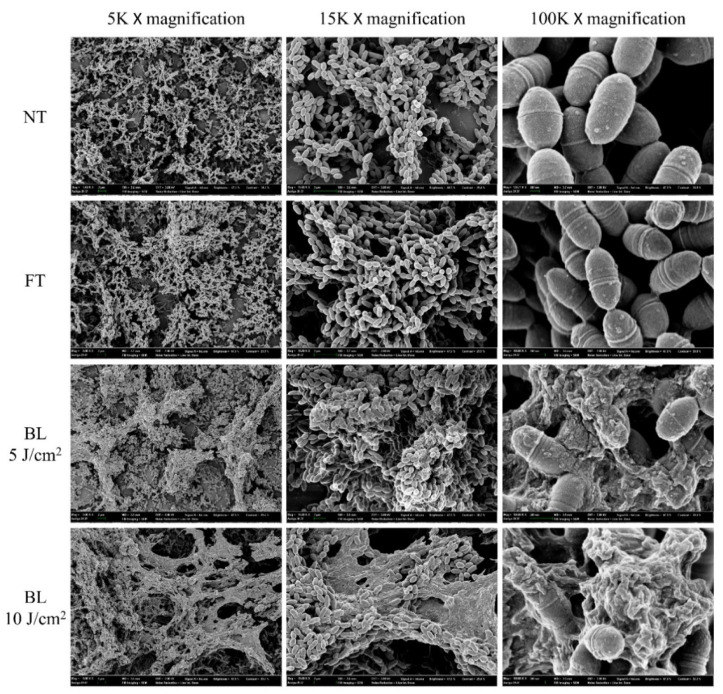
Field emission scanning electron microscope images of *Streptococcus mutans* biofilm treated with photodynamic therapy of fagopyrin F-rich fraction (FFF; final concentration of 5 µg/mL) exposed to blue light (BL). NT: *S. mutans* biofilm with no FFF treatment nor irradiation. FT: *S. mutans* biofilm treated with FFF without irradiation.

FE-SEM was used to visualize the PDI effect of FFF against *S. mutans* biofilms (Figure 5). Cell division in *S. mutans* occurs toward poles of cells and tends not to completely separate; hence the bacteria form chain shapes as they grow [31]. Before the PDI treatment as in the NT and FT, most *S. mutans* looked like short-chains in the biofilm (Figure 5). Namely, *S. mutans* formed an intact original shape without cell damage. However, after the PDI treatment of FFF, *S. mutans* cell membranes were destroyed by ROS produced from FFF (Figure 5). These FE-SEM images indicate that the cell membrane, not the intracellular cytoplasm, might be the major site of the damage mediated by ROS derived from FFF. Cell membrane destruction and cytoplasm leakage were clearly observed in the FE-SEM images at 15,000× and 100,000× magnifications, respectively (Figure 5). Also, *S. mutans* chain structures collapsed, resulting from cell membrane destruction by ROS from the PDI treatment of FFF. The FE-SEM images also showed a vast *S. mutans* biofilm destruction, as well as significant morphological changes in *S. mutans* chains after the PDI of FFF. This *S. mutans* biofilm destruction was energy fluence-dependent. Briefly, biofilms after the PDI treatment of FFF exposed to BL of 10 J/cm^2^ were more morphologically destroyed than BL of 5 J/cm^2^ (Figure 5). These data were consistent with the results described in Section 2.3. Also, the FE-SEM images of *S. mutans* biofilm destroyed by the PDI in this study were similar to those presented in the previous studies [21,23].

In general, ROS produced from PDI destroys cell membranes and denatures cellular DNA. Takasaki et al. reported that a PS destroys cell membranes via ROS production after attaching to the cell membrane rather than destroying DNA by intracellular ROS production [32]. ROS produced by PS membrane attachment inactivates the membrane transport system and associated enzymes, thereby inducing lipid peroxidation, which damages the cell membrane structure [32,33]. Esmatabadi et al. reported that after PDI-mediated cell membrane destruction, cytoplasm contents and metabolites were released from cells, and DNA was damaged [34]. In this study, we observed the destruction and cytoplasmic release of *S. mutans* cell membranes via the PDI treatment of FFF. Similar to other PS, FFF is believed to selectively bind to cytoplasmic membrane components and cause direct cell death by ROS via destruction of the membrane.

## 3. Materials and Methods

### 3.1. Chemicals and Reagents

Brain heart infusion (BHI) broth was purchased from BD (Becton, Dickinson and Company, Franklin Lakes, NJ, USA). ABDA, acetic acid, agar, crystal violet solution, DCFH-DA, D-mannitol, dihydroethidium (DHE), ethanol, formic acid, glycerol, and sodium azide were purchased from Sigma-Aldrich Co. (St. Louis, MO, USA). Glutaraldehyde, hexamethyldisilazane, osmium tetroxide, paraformaldehyde, and sodium cacodylate buffer (SCB) were purchased from Electron Microscopy Science (EMS) (Hatfield, PA, USA). Methanol and acetonitrile were purchased from J.T. Baker (Phillipsburg, NJ, USA). Phosphate buffer saline (PBS) and LIVE/DEAD™ Bacterial Viability Kit (L-7012) were purchased from Thermo Fisher Scientific (Waltham, MA, USA).

### 3.2. Bacterial Strain and Culture

*S. mutans* KCTC 3298 from the Korean Collection for Type Cultures (KCTC, Jeongeup, Korea) was cultured in the BHI broth at 37 °C. *S. mutans* stock solution was prepared by inoculating a single colony of bacteria from BHI agar plate into BHI broth (10 mL) and incubating for 24 h. After incubation, equal volumes of aliquoted bacterial broth of *S. mutans* and 50% glycerol were mixed, and the mixture was stored at −80 °C as stock. For subsequent experiments, the thawed *S. mutans* stock was inoculated into BHI broth (1%) and was cultured for 24 h. The cultured suspension of *S. mutans* was inoculated into fresh BHI broth (1%) and cultured for 24 h. The activated *S. mutans* (1.1 × 10^9^ CFU/mL) was used in subsequent experiments.

### 3.3. Preparation of FFF and Light Sources

TBF was collected as described in the previous study [9]. Freeze-dried TBF powder (500 mg) was extracted with methanol (20 mL) in a water bath (BS-11, Lab Companion, Seoul, Korea) at 60 °C for 60 min. The extract was centrifuged at 1500× *g* for 10 min. The supernatant was filtered through a 0.2 µm syringe filter (DISMIC-13JP, Advantec, Tokyo, Japan) and the filtrate was exposed to fluorescent light (400–700 nm; LED Bulb 12 W, Philips Korea, Seoul, Korea) for 2 h to convert protofagopyrins to fagopyrins. An HPLC system (e2695, Waters, Milford, MA, USA) equipped with a photodiode array detector (2998, Waters, USA) and an Agilent Zorbax Eclipse XDB (250 mm × 4.6 mm, 5 μm; Agilent, Palo Alto, CA, USA) column was used to separate FFF from the TBF extract. Mobile phases were 0.1% formic acid in distilled water (A) and 0.1% formic acid in acetonitrile (B). Flow rate was 1.2 mL/min. Gradient elution was set as follows: 60% B in 0–2 min, 60–100% B in 2–8 min, 100% B in 8–9.5 min, 100–60% B in 9.5–10 min, and 60% B in 10–11 min. Column temperature was 50 °C. Injection volume was 100 μL. The fraction (FFF) eluted between 9.0 and 10.0 min of retention time, at which fagopyrins detected at 590 nm were collected mostly, were dried using a centrifugal vacuum concentrator (VC 2200, Labogene, Seoul, Korea). The FFF powder was dissolved in methanol and stored at −80 °C for subsequent experiments. FF accounted for 92.33 ± 0.08% (*n* = 3) in the FFF, when calculated based on the peak areas obtained by HPLC-PDA and UPLC-MS/MS [3].

BL (450 nm; ABI 12 W Blue LED, ABI, Indianapolis, IN, USA), YL (590 nm; 15 W PI200, Bissol LED, Seoul, Korea), and RL (660 nm; ABI 12 W DEEP RED LED, ABI) were used in this study to find a suitable wavelength to activate FFF as a PS. Output powers of light sources were expressed as power density (W/cm^2^) and energy fluence (J/cm^2^), which were calculated as follows [23]:

Power density (W/cm^2^) = output power (W)/area (cm^2^)

Energy fluence (J/cm^2^) = power density (W/cm^2^) × exposure time (s)

### 3.4. ROS Production by FFF

#### 3.4.1. Intracellular ROS Production

Intracellular ROS produced in FFF-incorporated *S. mutans* suspension exposed to light was measured by fluorescence spectroscopy using DCFH-DA as described in previous studies with some modifications [20,27]. The cultured *S. mutans* suspension was diluted with PBS to 10^8^ CFU/mL. The suspension was incubated with DCFH-DA (final concentration of 5 μM) at 37 °C for 30 min in the dark. FFF (final concentration of 5 µg/mL) was added to the suspension, which was then treated with D-mannitol (type 1 ROS scavenger; final concentration of 100 mM) or sodium azide (type 2 ROS scavenger; final concentration of 100 mM) or not treated. The suspension was exposed to BL, YL, or RL (5 or 10 J/cm^2^). After the irradiation, fluorescence intensity was measured by SpectraMax iD3 (Molecular Devices, San Jose, CA, USA) at an excitation wavelength of 485 nm and an emission wavelength of 525 nm. Control was treated with the same final concentration of FFF without exposure to light.

#### 3.4.2. Superoxide Production

Intracellular superoxide produced in FFF-incorporated *S. mutans* suspension exposed to light was measured by fluorescence spectroscopy using DHE as described in previous studies with some modifications [35,36]. The cultured *S. mutans* suspension was diluted with PBS to 10^8^ CFU/mL. DHE (final concentration of 5 mM) was added to the suspension. The suspension was incubated at 37 °C for 30 min in the dark. FFF (final concentration of 5 µg/mL) was added to the suspension, which was then incubated at 37 °C for 15 min in the dark. After incubation, fluorescence intensity (excitation wavelength: 510 nm; emission spectrum: 560–650 nm) of the suspension was measured before exposure to light and every 10 s afterwards for 120 s while exposing the suspension to light (BL, YL, or RL) of 0.16 W/cm^2^. Fluorescence intensity was measured using SpectraMax iD3 (Molecular Devices). Control was treated with the same final concentration of FFF without exposure to light.

#### 3.4.3. Singlet Oxygen Detection

Singlet oxygen produced when FFF was exposed to light was measured by spectrophotometry using ABDA as described in a previous study with some modification [37].

ABDA (final concentration of 20 μM) and FFF (final concentration of 5 µg/mL) in PBS were added in 96-well plate (SPL, Pocheon, Korea). The absorbance spectrum (350–430 nm) was measured using SpectraMax iD3 (Molecular Devices). After measurement of the initial absorbance spectrum without exposure to light, it was measured every 10 s for 60 s while exposing the wells to light (BL, YL, or RL) of 0.16 W/cm^2^. Control was treated with the same final concentration of FFF without exposure to light.

### 3.5. Effect of S. mutans Inactivation with PDI Treatment of FFF

The effect of *S. mutans* inactivation with PDI treatment of FFF was performed as described in a previous study with some modification [21]. The PDI effect of FFF against *S. mutans* was determined using the plate count method. The cultured *S. mutans* suspension was diluted with PBS to 10^8^ CFU/mL. The suspension with FFF (final concentration of 2.5 or 5 μg/mL) was incubated at 37 °C for 10 min in the dark. Then suspensions were exposed to BL (5 or 10 J/cm^2^). After exposure, 10-fold serial dilutions in PBS were performed, and 100 µL of the suspensions were spread on BHI agar plates. After incubating for 48 h, the number of single colonies was counted. To figure out any intervention other than the PDI the following treatments were also tested: NT, FT, MT, and MIT.

### 3.6. Inhibition of S. mutans Biofilm Formation with PDI Treatment of FFF

Inhibition of *S. mutans* biofilm formation with PDI treatment of FFF was spectrophotometrically determined using crystal violet dye as described in the previous study with some modification [14]. The cultured *S. mutans* suspension was diluted to 10^8^ CFU/mL with fresh BHI broth containing 5% sucrose. The suspension (1 mL) was added to each well of a sterile and flat 24-well plate (SPL), and FFF (final concentration of 2.5 or 5 µg/mL) was added into the suspension. The suspension was incubated at 37 °C for 15 min in the dark. After incubation, the suspension was exposed to BL (5 or 10 J/cm^2^). The PDI-treated suspension was incubated to form biofilm at 37 °C for 24 h in the dark. The medium was decanted and gently washed twice with 1 mL PBS to remove loosely bound biofilm and unbound planktonic *S. mutans*. Biofilm was stained with 1 mL 0.1% crystal violet for 15 min on a well plate shaker (MX-M, DLAB, Riverside, CA, USA) at 300 rpm and room temperature (RT). The crystal violet dye was removed, and the biofilm was gently washed twice with 1 mL PBS. The stained biofilm was air-dried at RT for 15 min. After drying, 600 µL 33% acetic acid was added to dissolve the stained biofilm. The suspension was dissolved for 10 min on a well-plate shaker (DLAB, USA) at 300 rpm and RT. The absorbance of the dissolved biofilm suspension was measured using a SpectraMax iD3 (Molecular Devices) at 570 nm. Control was treated with the same final concentrations of FFF without exposure to BL. Biofilm formation level was expressed as the percentage of the control.

### 3.7. Visualization in PDI Effects of FFF against S. mutans Biofilm

#### 3.7.1. CLSM

The *S. mutans* suspension was diluted with fresh BHI broth containing 5% sucrose to 10^6^ CFU/mL. The suspension (3 mL) in a sterile confocal dish (SPL) was incubated at 37 °C for 24 h to form *S. mutans* biofilm. The medium was decanted and gently washed twice with 1 mL PBS to remove loosely bound biofilm and unbound planktonic *S. mutans*. One mL FFF (final concentration of 5 µg/mL) in PBS was added to the biofilm in the confocal dish. The suspension was exposed to BL (5 or 10 J/cm^2^). The suspension was removed to obtain the biofilm, which was then gently washed twice with 1 mL PBS. Biofilm was stained using LIVE/DEAD™ Bacterial Viability Kit (SYTO 9 and propidium iodide dye) according to the manufacturer’s instruction. The biofilm with staining solution was incubated in the dark at 37 °C for 1 h. The staining solution was removed and 100 µL PBS was added to prevent drying of biofilm. Fluorescence images were observed using CLSM (LSM710, Carl Zeiss, Oberkochen, Germany) at fluorescences of green (excitation wavelength: 488 nm; emission wavelength: 516 nm) and red (excitation wavelength: 543 nm; emission wavelength: 589 nm) under 40 times magnification. Control was treated with the same final concentrations of FFF without exposure to BL.

#### 3.7.2. FE-SEM

The *S. mutans* suspension was diluted to 10^6^ CFU/mL in fresh BHI broth containing 5% sucrose, and the suspension (3 mL) was incubated to form biofilm of *S. mutans* on glass coverslips (24 × 24 mm, Paul Marienfield, Lauda-Königshofen, Germany) in 6-well plates (SPL) at 37 °C for 36 h. After biofilm formation on the coverslip, the medium was decanted, and the well was gently washed twice with 1 mL of PBS to remove loosely bound biofilm and unbound planktonic *S. mutans*. One mL FFF (final concentration of 5 µg/mL) in PBS was added into each well, and the suspension was incubated at 37 °C for 5 min in the dark. After incubation, the suspension was exposed to BL (5 or 10 J/cm^2^). The suspension with FFF was decanted, and the biofilm on the coverslip was gently washed twice with 1 mL PBS. For primary fixation, the biofilm on the coverslip was soaked for 4 h in Karnovsky fixative containing 2% glutaraldehyde and 2% paraformaldehyde in 0.05 M SCB. The coverslip was washed three times with 0.05 M SCB for 5 min at each time. Post fixation was conducted using 1% osmium tetroxide in 0.1 M SCB at 4 °C for 1 h. After fixation, the coverslip was washed three times with distilled water for 5 min at each time and dehydrated in a series of ethanol solutions (30%, 50%, 70%, 80%, 90%, and 100%) for 10 min each. The coverslip was soaked in hexamethyldisilazane for 10 min for specimen drying, and then the coverslip was put in a vacuum desiccator for 24 h. After drying, the coverslip was mounted on stubs (EMS) and was coated with platinum by EM ACE 200 (Leica, Wetzlar, Germany). Biofilm images on the coverslip were obtained using FE-SEM (AURIGA, Carl Zeiss, Oberkochen, Germany). Control was treated with the same final concentrations of FFF without exposure to BL.

### 3.8. Statistics

All experiments were conducted in triplicate except for CLSM and FE-SEM. Statistical analysis was conducted by Student’s *t*-test and one-way analysis of variance (ANOVA) with Duncan’s multiple range test (*p* < 0.05) using SPSS 23.0 software (IBM, Armonk, NY, USA).

## 4. Conclusions

This study demonstrated that FFF when exposed to BL (450 nm) produced ROS, and the ROS production of FFF was on the basis of the type 1 mechanism. Furthermore, *S. mutans* and its biofilm were destroyed by ROS produced by PDI of FFF. It was elucidated that PDI with FFF (5 µg/mL) exposed to BL (10 J/cm^2^) destroyed *S. mutans* biofilms, which were visually confirmed by CLSM and FE-SEM. This study demonstrated for the first time that FFF, present in buckwheat plants, is a potent PS and can be applied to PDI. Although the PDI treatment of FFF was conducted at lower energy fluences of BL and lower concentrations than other PS, PDI effect of FFF against *S. mutans* was similar to curcumin and hypericin and was stronger than riboflavin. Thus, FFF might be a more effective PS for PDI against *S. mutans* than commonly used PS. Further studies would be needed to investigate whether the PDI of FFF can be applied to other gram-positive bacteria and tumors.

## Data Availability

Not applicable.
